# Learned Labels Shape Pre‐speech Infants’ Object Representations

**DOI:** 10.1111/infa.12201

**Published:** 2017-07-05

**Authors:** Katherine E. Twomey, Gert Westermann

**Affiliations:** ^1^ Department of Psychology Lancaster University

## Abstract

Infants rapidly learn both linguistic and nonlinguistic representations of their environment and begin to link these from around 6 months. While there is an increasing body of evidence for the effect of labels heard in‐task on infants’ online processing, whether infants’ learned linguistic representations shape learned nonlinguistic representations is unclear. In this study 10‐month‐old infants were trained over the course of a week with two 3D objects, one labeled, and one unlabeled. Infants then took part in a looking time task in which 2D images of the objects were presented individually in a silent familiarization phase, followed by a preferential looking trial. During the critical familiarization phase, infants looked for longer at the previously labeled stimulus than the unlabeled stimulus, suggesting that learning a label for an object had shaped infants’ representations as indexed by looking times. We interpret these results in terms of label activation and novelty response accounts and discuss implications for our understanding of early representational development.

Infants’ early‐acquired perceptual representations affect the way they respond to the world around them. For example, by three months, they have learned face representations which enable them to differentiate between own‐race and other‐race faces (Kelly et al., 2005). Similarly, just four months of experience of pets in the home are sufficient for infants to selectively attend to the most informative areas of animal stimuli in looking time tasks (Hurley & Oakes, 2015). This early representational development is powerful: in a two‐month training study, just two minutes’ experience per day with images of novel objects prompted five‐month‐old infants to learn representations which were sufficiently robust to affect their behavior in a later 3D object examining task presented at the end of the training period (Bornstein & Mash, 2010). Importantly, however, early learning is not just perceptual: in the early days, weeks and months infants also acquire linguistic representations. Even newborns can discriminate their native language from a nonnative language (Moon, Lagercrantz, & Kuhl, 2013) and detect grammatical categories in maternal speech (Shi, Werker, & Morgan, 1999). By eight months, infants can detect linguistic structure and segment words by tracking co‐occurrence statistics in the speech sounds they hear (Saffran, Aslin, & Newport, 1996).

Clearly, stored linguistic and nonlinguistic representations are linked—infants’ earliest words refer to the objects they experience on a daily basis (Clerkin, Hart, Rehg, Yu, & Smith, 2017). The first indications of these links appear before the onset of speech: infants as young as six months can correctly identify the referents of frequently heard words (Bergelson & Swingley, 2012; see also Delle Luche, Floccia, Granjon, & Nazzi, 2016). These early label‐object associations are strengthened incrementally over the long‐term via cross‐situational learning (Smith & Yu, 2008), in which repeated encounters of label‐object co‐occurrences in a variety of contexts eventually lead to long‐term word learning.

Importantly, these stored label‐object representations can shape online processing. For example, the structure of infants’ early vocabulary affects how they generalize category labels in‐the‐moment: toddlers whose vocabulary is dominated by count nouns which refer to solid objects in shape‐based categories show a strong tendency to generalize new nouns based on the shape of their referents, while this bias is reduced for children with a large number of nouns that do not follow this pattern (Perry & Samuelson, 2011). Equally, labels heard in‐the‐moment also begin to exert a powerful influence on processing during the first year. For example, the in‐task presence of a novel label can direct ten‐month‐old infants’ attention to commonalities between category exemplars and guide online category formation (Althaus & Plunkett, 2015; Plunkett, Hu, & Cohen, 2008), and labels themselves facilitate the formation of new representations over other auditory cues (e.g., Althaus & Westermann, 2016; for a review, see Robinson, Best, Deng, & Sloutsky, 2012).

Whereas it has been shown that both learned and novel linguistic representations affect infants’ nonlinguistic processing in‐the‐moment, it is not clear how linguistic experience shapes infants’ learned nonlinguistic representational structure. In adults, learned language has repeatedly been shown to shape representation in a range of perceptual domains, for example color, shape, and music (Dolscheid, Shayan, Majid, & Casasanto, 2013; Lupyan, 2016; Winawer et al., 2007). There is some evidence for similar effects in older children: in a target detection task in which a colored target was presented either on a same‐ or different‐color‐category background, toddlers who knew the relevant color labels detected targets more quickly in the left visual field, in line with adults in similar tasks. However, toddlers still learning color terms detected targets more quickly in the right visual field, suggesting that language learning may shape early perceptual representations, in the color domain at least (Franklin et al., 2008).

To our knowledge, only a single study has explicitly explored the relationship between learned labels and nonlinguistic representations in infants. Gliga, Volein, and Csibra (2010; E2) trained infants with novel 3D objects, labeling one (*Look at the blicket!*) and not the other (*Look at that!*) in a four‐minute play session. Immediately following training infants were presented with images of the two trained objects and a third novel object, while their neural responses were recorded via EEG (electroencephalograpy). Gamma‐band activity, which has been interpreted as a neurophysiological marker of object encoding, was significantly stronger in response to the labeled object than to the unlabeled or novel object, suggesting that labeling modulated infants’ object representations. However, it is unlikely that the training provided was sufficient for these 12‐month‐olds to retain the novel word over an extended period (Horst & Samuelson, 2008), and it is therefore possible that the task tapped temporary representations held in short‐term memory. Thus, whether or not infants’ learned language shapes their nonlinguistic representations remains unclear.

The following sections describe a test of this hypothesis in pre‐speech infants. We asked parents of ten‐month‐olds to train their infants with two novel toy objects at home over a week, labeling one object with a novel word (*labeled* object), but not the other object (*unlabeled* object). After this week‐long training, we recorded infants’ looking times in a familiarization task where they were shown both objects *in silence*. As it is long‐established that infants’ looking times in familiarization tasks reflect the characteristics of their learned representations (Fantz, 1964), an effect of language on infants’ long‐term object representations should be indexed by differences in looking times between the previously labeled and the previously unlabeled object. This being so, infants who had learned robust label‐object associations should show the effect most strongly. Thus, we also included a single preferential looking trial in which both images appeared simultaneously, accompanied by the label, and used infants’ responses on this trial as a proxy for this learning. This trial was included after familiarization to prevent the presentation of the label from biasing infants’ responses in the critical familiarization phase.

## Methods

### Participants

Twenty‐four ten‐month‐old infants (12 girls; *M*_age_ = 10 months, 23 days; *SD *= 14.15 days, range = 9 months, 26 days–11 months, 13 days) participated. All infants were typically developing and monolingual English learning with no family history of color blindness. Data from an additional six infants were excluded due to failure to start or complete the eyetracking task because of excess movement and/or crying (2); experimenter error (1), low eyetracker sample rate (<35%; 1); and failure to complete sufficient training sessions (2). All participants returned for the test session approximately a week after the introductory session (6 days: 2; 7 days: 19; 8 days: 3). Families were recruited by contacting caregivers who had previously indicated interest in participating in child development research. Caregivers’ travel expenses for both visits were reimbursed, and infants were given a storybook for participating.

### Stimuli

#### Play sessions

**Figure 1:**
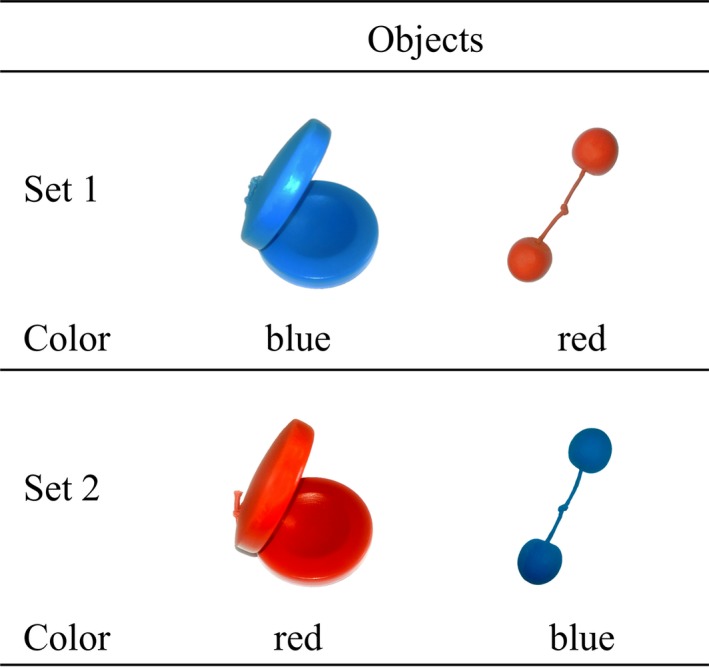
Stimuli used in the current study.

Three‐dimensional stimuli are depicted in Figure 1 and consisted of two age‐appropriate wooden toy objects (castanets and two wooden balls joined with string), chosen because they are novel to ten‐month‐old infants (Fenson et al., 1994). Objects were approximately equal in size and were painted either red or blue using nontoxic paint. The label was *tanzer*, a pseudoword selected because it is plausible in English and was used in a previous developmental study (Horst & Twomey, 2012).

#### Looking time task

Familiarization stimuli were digital photographs of the individual training objects presented centrally on a white background. Stimuli for the preferential looking trial were photographs of the training objects presented side‐by‐side on a white background. The auditory stimulus for the preferential looking trial consisted of the phrase *Look! A tanzer!* spoken by a female speaker from the local area and recorded and edited for timing and clarity in Audacity 2.0.6. The phrase onset was at 4,000 msec, label onset at 5,171 msec, and label offset at 6,000 msec. Calibration and attention getter stimuli were a short video of a bouncing cartoon bird, accompanied by a jingling sound.

### Procedure and design

#### Visit 1: Play session

Each infant received two objects. Objects’ color and label were counterbalanced between participants such that each infant received one red item and one blue item. Each exemplar was labeled for half of the infants and unlabeled for the other half of the infants.

First, the experimenter showed the caregiver the two objects and asked them whether their child had similar toys at home. Substitute items were available; however, no child had prior experience of the objects. The experimenter then explained that she would demonstrate a play session, with the goal of teaching the infant a word for one of the objects. She then asked the caregiver to conduct a similar play session for five minutes, every day for one week, and explained that they would be invited to return to the laboratory after seven days to take part in a looking time study.

The play session took place in a quiet, infant‐friendly room with the caregiver present at all times. Before the session began, the experimenter emphasized that caregivers should not invent a name for the unlabeled object: only the label *tanzer* should be used, and only in reference to the labeled object. With the parent watching, the experimenter then sat opposite the infant on the floor and introduced both toys by holding them in front of the infant and allowing the infant to take the toys in their own time. While the infant was looking at the toy, the experimenter referred to them using a label or a pronoun as appropriate, for example “*Look, a tanzer!*” (labeled), “*Look at this!*” (unlabeled). The experimenter explained to the caregiver that they should encourage their child to interact with both toys for an approximately equal amount of time, and that their child should be allowed to play with both toys at the same time (to encourage comparison, which promotes encoding; Gentner & Namy, 1999; Oakes, Kovack‐Lesh, & Horst, 2009). Infants heard the label approximately twice every 15 seconds. After the play session, caregivers were given the toys, written instructions and a sticker chart on which to record their play sessions.

#### Visit 2: Looking time task

Before the second session began, caregivers were asked whether they had completed all play sessions. All but three parents reported completing a play session on all seven days. Verbal report tallied with the sticker charts (7 sessions: 21; 6 sessions: 3). Parents reported no difficulty in completing the sessions, although some reported an overall decline of interest in the stimuli by the end of the week.

The looking time task took place in a quiet, dimly lit testing room. Children were seated on their caregiver's lap 50–70 cm in front of a 21.5″ 1,920 × 1,080 computer screen. A Tobii ×120 eyetracker located beneath the screen recorded the child's gaze location at 17 msec intervals, and a video camera above the screen recorded the caregiver and child throughout the procedure. Caregivers were instructed not to interact with their child or look at the screen during the task to avoid biasing their child's behavior.

The eyetracker was first calibrated using a five‐point infant calibration procedure. We displayed an attention‐grabbing animation in the four corners and center of a 3 × 3 grid on a gray background accompanied by a jingling noise, and recorded infants’ orientation to it with a key press. Calibration accuracy was checked and repeated if necessary (one infant).

The attention‐getting stimulus then appeared in the center of the screen. Immediately after the infant oriented toward the attention getter, the experimenter began the familiarization phase using a keypress. Familiarization stimuli were presented individually in silence for ten sec. Infants saw eight identical images of the previously labeled object and eight identical images of the unlabeled object. Presentation of both objects was interleaved. The object shown first was counterbalanced between children. Each trial was immediately followed by the attention getter. Subsequent trials were advanced manually by the experimenter once the infant had reoriented to the screen, or began automatically after five sec.

Immediately following the familiarization trials, a single preferential looking trial was presented in an identical manner. Left–right positioning of the objects (castanet/ball and labeled/unlabeled) was counterbalanced between children. The preferential looking trial was 12 sec long, with auditory stimulus beginning at 4,000 msec, label onset at 5,171 msec and offset at 6,000 msec.

### Coding and data cleaning

Time stamps for which the eyetracker failed to reliably detect either eye were excluded (41.06%; this is broadly in line with existing studies of data reliability in infant eyetracking work; Wass, Smith, & Johnson, 2012). On each familiarization trial, the area of interest (AOI) was centered on the single image and measured approximately 950 by 700 pixels. On the preferential looking trial, AOIs divided the screen in half horizontally and were centered vertically, measuring 980 by 860 pixels. Individual gaze samples were numerically coded (−1 = look away, 0 = background look, 1 = AOI look), creating a raw looking time measure. For familiarization trials, looks away from the screen were discarded (16.31%) and for preferential looking trials, non‐AOI looks were discarded (0.08%). This resulted in a final dataset of 89,099 familiarization trial and 13,213 preferential looking trial gaze samples.

## Results

If learned labels shape infants’ long‐term object representations, we hypothesized that infants who had learned an association between the label and the corresponding stimulus should exhibit differences in looking times when viewing the previously labeled vs. the previously unlabeled stimulus, even when these stimuli were presented in silence. Thus, our primary variable of interest was looking times during the familiarization phase. However, on this account, infants with more robust label associations should show greater differences in looking time. Thus, we first analyzed the preferential looking trial to obtain an index of individual infants’ label responses as a proxy for the strength of their label‐object associations.

In looking time studies employing the habituation paradigm, an increase in looking to a novel stimulus after the habituation phase is taken as an indicator that infants are attending to the task, allowing researchers to rule out fatigue as a cause of any subsequent effects (Oakes, 2010). It is possible that fatigue could affect children's looking times in this study, particularly as we employed a fixed duration familiarization phase rather than an infant‐controlled habitation phase. To rule out the influence of fatigue on infants’ preferential looking, we compared their prelabel looking on the preferential looking trial to their looking on the final trial of the previous familiarization phase. Specifically, we defined a prelabeling block as the 5,171 msec before the label onset, and a final‐trial block as the final 5,171 msec of the final familiarization trial. For both blocks, we calculated each infant's proportion of looking to the AOI out of total screen looks, and submitted these proportions to a two‐tailed paired samples *t*‐test. The *t*‐test confirmed that infants’ responses to the label on the preferential looking trial were unlikely to be the result of fatigue (*t*(21) = 2.65, *p *=* *.015): Infants’ proportion AOI looking was greater for the prelabeling block (*M *=* *0.99, *SD* = 0.04) than at the end of the final familiarization trial (*M *=* *0.92, *SD* = 0.11).

**Figure 2:**
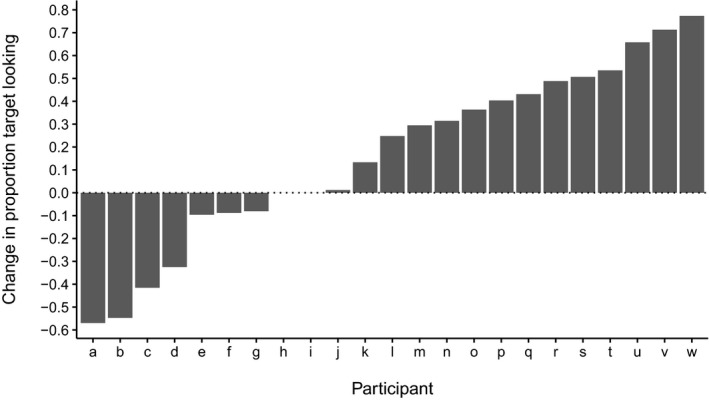
Individual infants’ change in proportion target looking from the pre‐ to the post‐labeling phase.

Next, to obtain an index of infants’ responses to the label, we defined a post‐labeling time window between 233 and 2,000 msec after label onset (Mani & Plunkett, 2010) and a corresponding pre‐labeling window as the 2,000 msec immediately preceding label onset. Twenty‐one infants contributed data to the pre‐labeling window and 23 to the post‐labeling window. To establish whether infants responded to the label, we conducted two analyses. First, we examined overall changes in proportion of target looking. Infants showed no evidence of a pre‐labeling target preference (*M *=* *0.50, *SD* = 0.30, *d *=* *.0069; *t*(20) = −0.032, *p *=* *.98; all tests two‐tailed). Post‐labeling, infants’ small preference for the target (*M *=* *0.62, *SD* = 0.33, *d *=* *.36) did not reach significance (*t*(22) = 1.73, *p *=* *.098). However, infants overall showed a small increase in target preference from pre‐ to post‐labeling (*d *=* *0.42), although this difference was not robust (*t*(22) = 2.00, *p *=* *.058). Next, to obtain individual response scores, we subtracted each infant's pre‐ from post‐labeling proportion target looking (Bergelson & Swingley, 2012). Scores are depicted in Figure 2. While some infants incorrectly switched from looking at the target to the distracter after labeling (infants *a*–*g*), and some showed no response to the label, infants *j*–*w* correctly increased their target looking, in some cases substantially. Thus, inasmuch as these shifts in attention serve as an index of having learned the label (we return to this issue in the Discussion), this analysis suggests that at least some infants had learned a label‐object association sufficiently robust to allow them to correctly shift their attention to the target. Critically, if learned labels affect infants’ object representations, those infants who responded correctly should also show greater differences in looking times in the preceding silent familiarization phase. We therefore incorporated these response scores as a predictor in our main analysis of looking times during familiarization.

**Figure 3:**
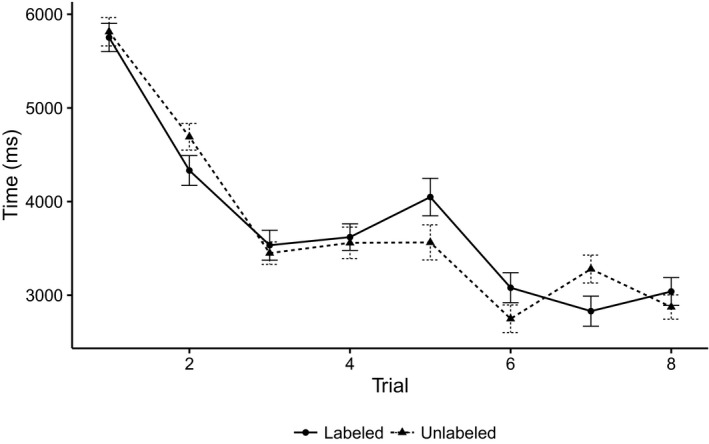
Looking times to labeled and unlabeled stimuli during familiarization. Error bars represent 95% confidence intervals after removal of random errors from the model (Hohenstein & Kliegl, 2014).

Overall looking times during familiarization are depicted in Figure 3. We submitted infants’ looks to the stimulus (i.e., AOI looks) to a binomial mixed effects model using the R package lme4 (version 1.1‐11; Bates, Mächler, Bolker, & Walker, 2015). We included fixed effects of label (labeled = 1, unlabeled = 0), trial (1–8) and response score and their two‐way interactions. The three‐way interaction was dropped to achieve model convergence. Random effects were selected by fitting a maximal random effects structure and simplifying until the model converged (Barr, Levy, Scheepers, & Tily, 2013). The final model included by‐participant intercepts. Results are presented in Table 1.

**Table 1:** Results of Mixed Effects Model

	*β*	*SE*	*z*	*p*
Label	0.23	0.071	3.22	.0013**
Trial	−0.06	0.0098	−6.19	<.001***
Response	0.95	0.44	2.18	.029*
Label × trial	−0.019	0.014	−1.34	0.18
Label × response	−0.56	0.081	−6.90	<.001***
Trial × response	−0.078	0.017	−4.53	<.001***

*p < .05, **p < .01, ***p <  .001

As is typical in looking time studies, infants became less likely to look toward the stimulus as familiarization progressed (negative main effect of trial). While this decrease was not different for the labeled or unlabeled stimulus (nonsignificant label by trial interaction), the odds of looking to the stimulus decreased faster for infants with higher response scores than infants with lower response scores (negative trial by response interaction). Higher‐response infants were also more likely to look at the stimulus overall (positive main effect of response). Importantly, infants were overall more likely to look at the labeled than the unlabeled stimulus. This supports our main hypothesis: whether infants had previously been taught a label for an object affected their looking times (positive main effect of label). Furthermore, this label effect interacted with infants’ response scores.

**Figure 4:**
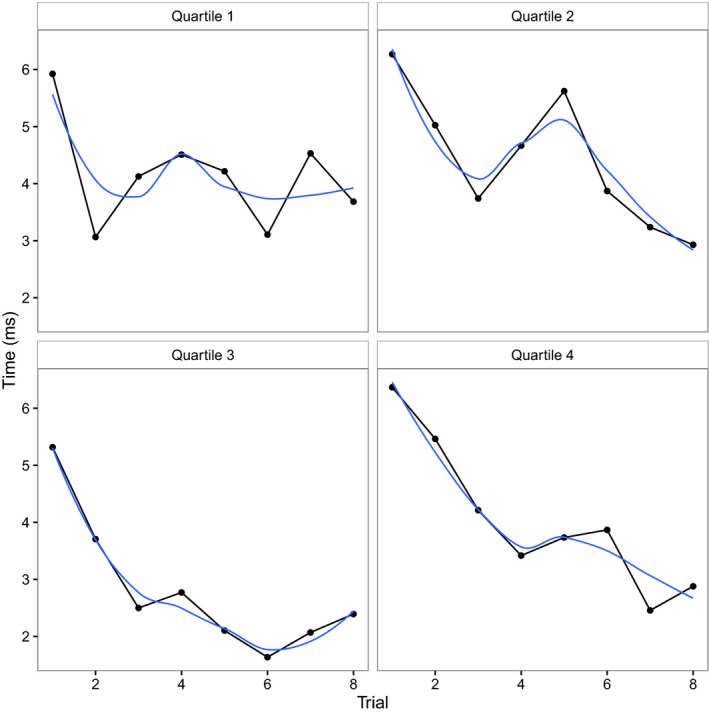
Looking times to labeled stimulus, split by response score quartiles. Blue line represents loess smoothing performed in the R package ggplot2 (Wickham, 2016).

To understand this interaction, we ran two separate binomial mixed effects models on raw looking times to the previously labeled and unlabeled stimuli, each with fixed effects of trial and response score and their interaction, and retaining the same random effects structure as the previous model. When infants viewed the unlabeled stimulus, they were less likely to look at the stimulus across trials (main effect of trial: *β* = −0.067, *SE* = 0.010, *z *=* *−6.73, *p *<* *.001). Response scores had no effect (main effect: *β* = 0.70, *SE* = 0.55, *z *=* *1.26, *p *=* *.20; trial × response score: *β* = 0.030, *SE* = 0.026, *z *=* *1.13, *p *=* *.26). Thus, whether infants had responded correctly or incorrectly to the label after familiarization, their odds of looking at the unlabeled stimulus were the same. When infants viewed the previously labeled stimulus, they were also less likely to look at the stimulus across trials (main effect of trial: *β* = −0.079, *SE* = 0.010, *z *=* *−7.52, *p *<* *.001). Response scores had no independent effect (main effect: *β* = 0.57, *SE* = 0.46, *z *=* *1.26, *p *=* *.21). However, there was an interaction between the effect of response score and trial on infants’ odds of looking at the stimulus (*β* = −0.15, *SE *= 0.023, *z *=* *−6.47, *p *<* *.001). Because this interaction involved two continuous variables, to explore it, we grouped children by response score percentiles and plotted them. As shown in Figure 4, although the relationship between response score and looking time is complex, infants with highest response scores initially looked for longest at the labeled object and showed a steep, relatively smooth decline in looking, while infants with lower response scores showed a more variable profile with a shallower decline. Overall, however, learning a label for an object did affect infants’ looking times to that object, even when presented in silence.

## Discussion

The current study explored whether infants’ learned linguistic representations influence their learned nonlinguistic representations. Ten‐month‐old infants were trained over a week by their caregivers with two 3D objects and were taught a novel label for just one of them. When these objects were presented in silence in a looking time task, infants spent longer looking at the previously labeled than the unlabeled stimulus. Further, whether or not infants responded to the label on a final preferential looking trial affected their looking times—but only when viewing the previously labeled stimulus. Given that training and familiarization for each object were identical except for the presence of the label during the play sessions, taken together these finding suggest that prior label training affected infants’ responses in the silent looking time task.

While infants looked for longer overall at the labeled stimulus during familiarization, we found differences in looking between infants who responded correctly to the label on the preferential looking trial and those who responded incorrectly. Importantly, latency to respond to labeling may provide an index of infants’ speed of verbal processing, rather than depth of lexical representation (Fernald, Pinto, Swingley, Weinbergy, & McRoberts, 1998). Thus, it is possible that our response scores measure intrinsic individual differences rather than whether or not infants had learned the word. However, these contrasting patterns of looking emerged in response to the previously labeled stimulus only; if our response scores tapped some phenomenon unrelated to infants’ strength of label‐object representations, we would expect similar differences to emerge in response to the unlabeled stimulus. Nonetheless, not all infants responded correctly to the label, and some shifted their attention away from the target. We do not therefore claim that these prespeech infants learned a new word during training, but rather we interpret these results as supporting accounts of early word learning in which infants learn label‐object associations incrementally (Bion, Borovsky, & Fernald, 2013; McMurray, Horst, & Samuelson, 2012; Yurovsky, Fricker, Yu, & Smith, 2014). That is, these infants learned something about the object, something about the label, and something about the mapping between the two. These partial associations were then sufficient to influence infants’ looking times. Overall, while future research is needed to delimit the boundaries of very young infants’ word learning abilities, this study suggests that 10‐month‐old infants are capable of learning at least partial label‐object mappings from limited exposure.

### Mechanism

Critically, the familiarization phase was silent, and which object had been labeled during training was counterbalanced across participants. Thus, infants’ longer looking times to the labeled object could only have arisen due to some kind of difference in their representations of the two objects, and these differences can only have been due to the presence of a label during training. Two mechanisms could account for these results: label activation, or novelty preference.

Existing research demonstrates that infants will interact for longer with objects in the presence of those objects’ labels (Baldwin & Markman, 1989). If seeing an object activates its label representation, then this activation could in turn trigger increases in looking times. Indeed, implicit naming of silently presented images has been demonstrated in 18‐month‐old infants (Mani & Plunkett, 2010). This label activation account is compatible with theories of representational structure in which labels and objects are represented separately, either qualitatively differently (Waxman & Markow, 1995) or distantly in the same representational space (Westermann & Mareschal, 2014), and become linked over experience. On these accounts, linguistic representations are separate from nonlinguistic representations, but affect them through association. Alternatively, the observed looking time differences could reflect a novelty preference. Specifically, several “labels‐as‐features” accounts of early representational development assume that labels initially serve as one among multiple nonreferential features in object representations (Gliozzi, Mayor, Hu, & Plunkett, 2009; Sloutsky & Fisher, 2004; Sloutsky & Lo, 1999); for example, the word *strawberry* and the color red will have the same status in a speaker's representation of the fruit. Thus, if a stored representation *incorporates* a label, then encountering the object without the label results in an incongruent online representation (Lupyan, 2008). This incongruence evokes a novelty response—indexed in the current study by increased looking times during familiarization to the previously labeled object.

While it is not possible to ascertain which of these two accounts are the most plausible in the context of the data presented here, each account makes testable predictions, pointing to future studies to help delineate between the two. First, the implicit naming account requires an extension of Mani and Plunkett's (2010) lexical priming effects on 18‐month‐old toddlers to 10‐month‐old infants: if younger infants do not activate learned labels when encountering their referents in silence, we would expect no differences in looking time during familiarization in our study. Second, on “labels‐as‐features” accounts, if a label is shared between multiple exemplars of a category, this shared feature should increase between‐exemplar similarity (see also Westermann & Mareschal, 2014). Thus, with the current design, training infants with a category of labeled objects and a category of unlabeled objects should provoke a novelty preference during familiarization for the previously *unlabeled* object. Finally, computational work which explicitly models these two accounts is currently underway (Capelier‐Mourguy, Twomey, & Westermann, 2016).

More broadly, the current study contributes to our understanding of the relationship between early language learning and representational development. We demonstrate that prespeech infants can learn label‐object associations in just one week that are sufficiently robust to affect their subsequent looking times to these objects when presented in silence. These findings offer converging evidence that learning a label for an object restructures that object's representation, and in turn affects behavior in‐the‐moment, illustrating the multiple timescales at play in early representational development.
